# Ten-Year Follow-Up of Desarthrodesis of the Knee Joint 41 Years after Original Arthrodesis for a Bone Tumor

**DOI:** 10.1155/2015/308127

**Published:** 2015-11-25

**Authors:** Ahmed Hamed Kassem Abdelaal, Norio Yamamoto, Katsuhiro Hayashi, Akihiko Takeuchi, Shinji Miwa, Hiroyuki Inatani, Hiroyuki Tsuchiya

**Affiliations:** ^1^Department of Orthopedic Surgery, Graduate School of Medical Science, Kanazawa University, 13-1 Takaramachi, Kanazawashi, Ishikawa Prefecture 920-8641, Japan; ^2^Department of Orthopedic Surgery, Faculty of Medicine, Sohag University Hospital, Nasser city, Sohag 82524, Egypt

## Abstract

*Introduction*. The main indication for knee arthrodesis in tumor surgery is a tumor that requires an extensive resection in which the joint surface cannot be preserved. We report a patient that had knee desarthrodesis 41 years after giant cell tumor resection followed by a knee arthrodesis. This is the longest reported follow-up after desarthrodesis and conversion to total knee arthroplasty (TKA), almost ten years.* Case Report*. A 71-year-old man with a distal femoral giant cell tumor had undergone a resection of the distal femur and knee arthrodesis using Kuntscher nail in 1962. In July 2003 he experienced gradually increasing pain of his left knee. We performed a desarthrodesis and conversion to TKA in 2005. The postoperative period passed uneventfully as his pain and gait improved, with gradually increasing range of motion (ROM) and no infection. He now walks independently, with no brace or contractures.* Conclusion*. Desarthrodesis of the knee joint and conversion to TKA are a difficult surgical choice with a high complication risk. However, our patient's life style has improved, he has no pain, and he can ascend and descend stairs more easily. The surgeon has to be very meticulous in selecting a patient for knee arthrodesis and counseling them to realize that their expectations may not be achievable.

## 1. Introduction

Bone tumors around the knee frequently have been treated by resection arthrodesis since the last century. Lexer was the first to describe resection of bone tumors with subsequent arthrodesis of the knee joint in 1908 [1–3]. This encouraged more surgeons to use this procedure and report their results, which showed successful tumor control, but unfortunately with many complications that significantly affected rehabilitation. About half of the patients in early studies suffered complications and the incidence of subsequent amputation was high [4, 5].

The main indication for arthrodesis in tumor surgical treatment was a tumor that required an extensive resection in which the joint surface could not be preserved, but a safe posterior margin could be achieved without jeopardizing the neurovascular structures. Tumors that did not meet these criteria were best managed with amputation [3]. A previous contralateral knee joint arthrodesis and neuromuscular disease are contraindications for knee joint arthrodesis [2]. Enneking and Shirley performed knee arthrodesis to treat recurrent tumors around the knee in twenty patients. Fifteen of sixteen patients in their series resumed their previous occupations [6].

In our department, knee arthrodesis was used at that time with almost the same indication and techniques, until reconstruction procedures had been developed to include more options, that is, arthroplasty, composite allografts, distraction osteogenesis, and biological reconstruction by frozen bone autograft.

We report a case that had knee desarthrodesis 41 years after a primary arthrodesis for treatment of giant cell tumor. This is the longest reported follow-up after desarthrodesis and conversion to total knee arthroplasty (TKA), almost ten years.

## 2. Case Report

A 71-year-old man had presented to our department with knee pain and swelling in 1962 when he was 17 years old and was diagnosed with giant cell tumor. In October 1963 he underwent tumor resection and substitution by autogenous bone graft. In 1964 his tumor recurred, and he had a resection of the distal femur and knee arthrodesis using a Kuntscher nail, which was a popular technique for the management of bone tumors at that time.

**Figure 1:**
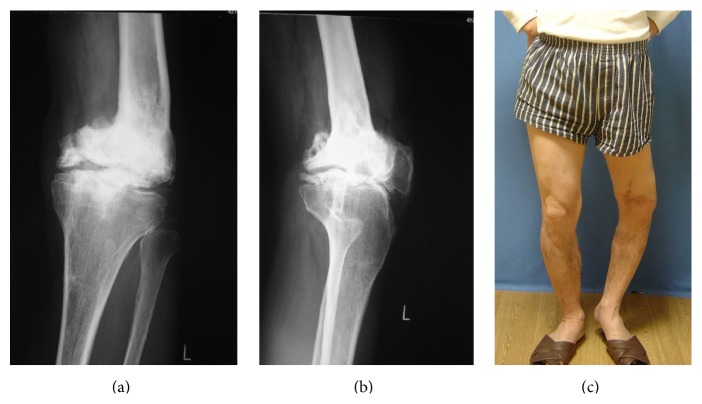
Preoperative condition. (a, b) Anteroposterior and lateral X-P (plain X-ray or roentgenography) of the knee showing partial pseudarthrosis of the knee and varus deformity of the joint. (c) Clinical photo of the patient shows marked bowing.

From 1964 until 2003, the patient was lost to follow-up, according to his history. He was not complaining of pain or any other knee symptoms until July 2003 when he experienced gradually increasing pain of his left knee, and a sense of instability. The pain and instability slowly increased despite the use of a knee brace. Clinical evaluation of the knee revealed minimal movement, that is, a state of pseudarthrosis, with obvious varus deformity. X-rays were taken and revealed marked arthritic changes in an almost pseudoarthrotic knee joint (Figure 1). Leg length discrepancy (LLD) was 5 cm. We discussed the treatment options with the patient, including refusion either with a bone graft or by distraction osteogenesis, or the challenging option of desarthrodesis and conversion to TKA. The patient chose the most difficult: to convert his failed knee arthrodesis by a TKA. We explained to him the possible complications which could include amputation.

**Figure 2:**
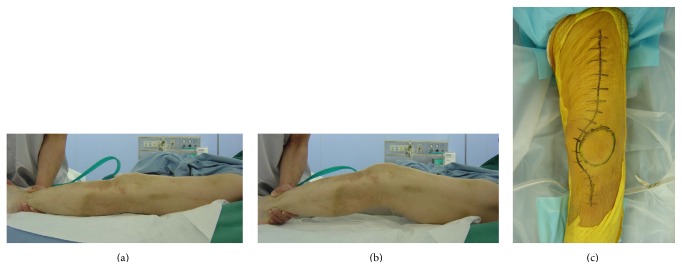
Preoperative planning. (a, b) Examination under anesthesia showing ROM 0–25°. (c) Anteromedial approach.

**Figure 3:**
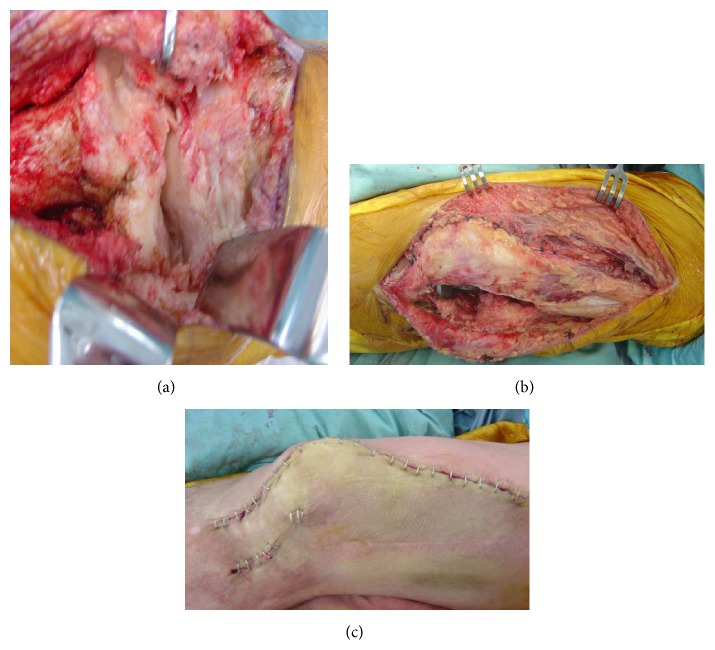
Intraoperative photos. (a) Pseudarthrosis of the arthrodesed knee joint. (b) Soft tissue closure, that is, repair of the medial patellofemoral ligament and release of the lateral retinaculum. (c) Skin closure after desarthrodesis, with a separate lateral incision for release.

In 2005, the surgery was performed under general anesthesia and excision of the arthrodesis via an anteromedial approach was carried out (Figure 2). Reconstruction was done using a Howmedica Modular Reconstruction System (HMRS) with correction of the LLD and the varus deformity. A lateral release incision was performed to release the lateral patellar retinaculum. Skin closure was feasible without tension (Figures 3(b) and 3(c)). There was blood loss of 330 mL, and the surgery took 3 hours and 35 minutes.

Immediately postoperatively, the knee joint did not show much improvement with an ROM about 0–40° due to stiffness of the knee joint. We did not try to mobilize the joint under anesthesia. A rehabilitation program started 48 hours after surgery, with emphasis on ROM exercises, muscle strengthening, standing load practice, and walking practice with partial weight bearing. Full weight bearing was allowed after four weeks. Pain improved after surgery, with gradually increasing ROM and improved gait. No infections occurred and the postoperative period passed peacefully.

**Figure 4:**
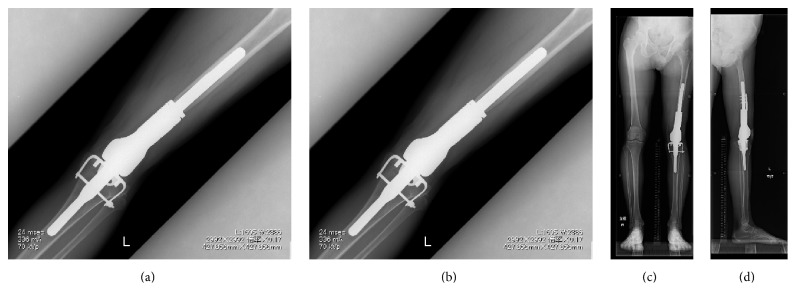
Last follow-up X-P. (a, b) X-P of the knee joint after arthroplasty, 10 years after surgery. (c, d) Whole leg standing X-P showing improvement of the mechanical axis and LLD.

**Figure 5:**
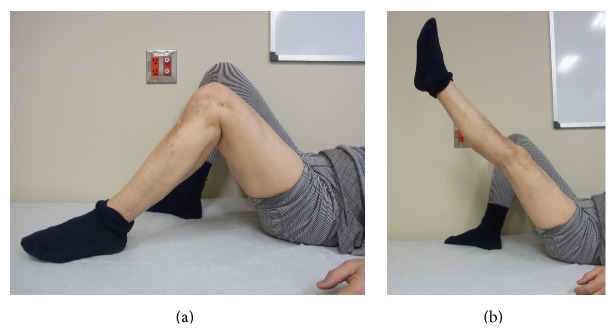
Last follow-up clinical photo. (a) Clinical photo in maximum flexion of the knee joint (about 100°). (b) Clinical photo in maximum extension of the knee joint (about 0°).

Up to the present, the patient visits our department regularly. His flexion ROM has improved to 90–100°, he has no pain, and he walks independently with no brace or contractures (Figures 4 and 5). The patient is satisfied and states he would have this operation again under the same circumstances.

The patient was informed about this case report and agreed to its publication. It also was approved by our IRB.

## 3. Discussion

Orthopedic surgeons are familiar with arthrodesis surgery, and for the knee joint they perform arthrodesis for many indications. The best-known justification for knee arthrodesis is to treat a painful, unstable knee in a young active patient. It has been performed for postpoliomyelitis syndrome, Charcot gonarthrosis, posttraumatic arthritis, and resistant infections and after the excision of bone tumors.

While the surgical outcome for a painful unstable knee following resection of the articular cartilage and fusion of the knee joint is generally positive, the outcome for a desarthrodesis knee is often negative, and there is a belief that the decision to perform this operation is rash.

The complication rate in conversion of an arthrodesis knee to TKA is very high (53%–57%). Complications include delayed wound healing and skin sloughing, quadriceps tendon rupture, patellar tendon rupture or residual extensor lag, and postoperative stiffness [7–9]. The possibility of a second surgery is also very high, either for treating infection, for rearthrodesis because of persistent pain, or a revision surgery for the loosening of the implant [7–14].

In addition, in tumor surgery, the arthrodesis procedure is primarily indicated when the tumor requires extensive quadriceps muscle resection and for those requiring extra-articular extension [2]. Therefore, there is more expected soft tissue resection than if it was done for other indications. This makes the mere suggestion of conversion to TKA a difficult option for the surgeon.

Though there are some studies that have investigated the conversion of a knee arthrodesis to TKA in a wide range of primary indications for the arthrodesis, studies of desarthrodesis and conversion to a TKA after bone tumor resection are scarce. Bradley et al. [10] reported their results of desarthrodesis of the knee after resection of bone tumors in 16 cases. Their results were not optimal; most of their patients had complications ranging from wound infection to local amputation of the limb as a result of recurrence. This is not surprising given that most of the patients had a wide marginal resection, which means more soft tissue resection and a more difficult task for reconstruction and postoperative rehabilitation. Moreover, these results encouraged them to emphasize that the procedure is highly demanding and carries a high risk of complication. Patients for the procedure should be selected very carefully.

Reconstructions by joint arthrodesis after bone tumor resection have declined to a great extent by virtue of new reconstruction options after bone tumor excision, especially in young adults for whom arthrodesis had been considered as a treatment of choice for a long time. Now a variety of techniques, extendable endoprosthesis, composite allografts, and biological reconstruction of bone by freezing, autoclaving, pasteurization, or irradiation, are all options for preserving the mobility of the joint rather than getting it arthrodesed. Furthermore, recent advances in the field of neoadjuvant chemotherapy have allowed the surgeon to excise the tumor with much less soft tissue sacrifice, leading to a more favorable functional outcome.

In this case report, the patient had a single recurrence one year after the primary resection surgery, but none later. His arthrodesis was sound for more than 39 years, when he started to complain about pain and instability. This might be explained by fatigue failure of the arthrodesis because of the length of time it had been in a physically active patient. This long period since the initial arthrodesis makes the decision for desarthrodesis riskier because of the expected soft tissue contracture or for fear of quadriceps tendon rupture. However, in view of the gradually aggravated symptoms of the patient, we felt the risk was justified; even revision surgery for arthrodesis has a risk for complications.

Knee arthrodesis can be achieved through an anteromedial or anterolateral approach [2]. In this case it was performed via an anteromedial approach. In the desarthrodesis procedure, we preferred to go through the previous incision to minimize the cosmetic effects of the procedure, but we performed a lateral release to allow the patella to fit optimally to the implant and avoid the possibility of patella baja due to the long-standing arthrodesis and contracture of the patellar tendon. The type of implant used also may affect the outcome. When there are deficient collateral ligaments with adequate bone stock, a constrained total knee prosthesis is better in the conversion of a fused knee to compensate the deficient collateral ligaments [8, 12]. However, Clemens et al. advocated use of semiconstrained implants [15], and Kim et al. [16] recommended a posterior stabilized implant, provided that the soft tissue sleeves can be preserved. After the tumor excision, there is always an expectation of inadequate bone stock as well as deficient collateral ligaments; in this situation it is better to use a constrained hinged prosthesis. In the present case we used HMRS (Howmedica International, Limerick, Ireland) which is a first-generation modular endoprosthetic system. This system has intramedullary cementless press-fit stems supported by external flanges and cortical transfixation screws, whereas the knee mechanism consisted of a simple hinge design [17]. In this case we report excellent results with this type of implant although Martin Malwer reported that long-term outcomes after use of this implant were disappointing. Significant problems were encountered with this device, including aseptic stem loosening (osteolysis), substantial stress shielding with bone resorption, screw fracture and migration, and a polyethylene failure rate higher than 40% for the knee mechanism [17–19]. The time interval between arthrodesis and the conversion to TKA is also expected to affect muscle function and strength. To overcome this, the posterior hinge of the prosthetic knee joint allows stabilization in hyperextension which requires lower muscle strength, and in cases of the absence of quadriceps function it enhances the stability of the knee in the stance phase of gait.

Several techniques have been suggested and used in an attempt to overcome the contracture of soft tissues. Cho et al. [20] reported a two-stage conversion of the fused knee to TKA. In the first stage, they implanted a soft tissue expander subcutaneously, to avoid problems of skin sloughing and wound dehiscence, and performed TKA as a second stage. Mahomed et al. [21] reported a two-stage use of two soft tissue expanders, one inserted subcutaneously, and the other inserted under the patella through a pocket in the vastus lateralis muscle. These expanders yielded good results, but there is a risk of complications, especially infection, which may threaten the success of the arthroplasty. Kim et al. [16] performed V-Y plasty of the quadriceps tendon to minimize the contracture and facilitate regaining of ROM. In our case, we examined the soft tissue around the knee and around the previous scar and determined that we would not face a problem with the soft tissue covering after the conversion procedure. Therefore, we did not use either of these previously described methods. It was sufficient to perform a separate lateral incision to release the tightness of the lateral patellar retinaculum to avoid lateral displacement of the patella after wound closure.

## 4. Conclusion

Desarthrodesis of the knee joint and conversion to TKA are a difficult choice operatively, with a high risk of complications, especially when the primary arthrodesis procedure is done after tumor resection. Nevertheless, our patient's lifestyle improved markedly, with no pain, and he can ascend and descend stairs more easily. He would have the operation again if he was in the same circumstances.

For patients being considered for a knee arthrodesis, especially if done for bone tumor, the surgeon has to be very meticulous in patient selection and counsel the patient to realize that their expectations may not be fully achievable.
